# Use of Generic Large Language Model Chatbots for Mental Health Issues Among Patients Seeking Psychotherapy: Cross-Sectional Mixed Methods Analysis in a German University Hospital Outpatient Clinic

**DOI:** 10.2196/104297

**Published:** 2026-10-09

**Authors:** Alexander Diel, Niels Weber, Jil Beckord, Tania Lalgi, Christoph Jansen, Eva-Maria Skoda, Nora Dörrie, Venja Musche, Anita Robitzsch, Martin Teufel, Alexander Bäuerle

**Affiliations:** 1 Clinic for Psychosomatic Medicine and Psychotherapy LVR University Hospital Essen University of Duisburg-Essen Essen, North Rhine-Westphalia Germany; 2 Center for Translational Neuro- and Behavioral Sciences University of Duisburg-Essen Essen, North Rhine-Westphalia Germany

**Keywords:** large language model, generative AI, mental health, chatbot, psychosomatic medicine, outpatient

## Abstract

**Background:**

Large language model–based chatbots (LLM-CBs) are increasingly used as mental health support tools. Risks and harms are discussed especially for unsupervised use and for vulnerable groups. A naturalistic characterization of the use of LLM-CBs by patients with mental health disorders is currently lacking.

**Objective:**

The goal of this cross-sectional study is to investigate demographic and clinical characteristics of users of LLM-CB for mental health and their associations with use behavior.

**Methods:**

Across 6 months, qualitative and quantitative data on LLM-CB use for mental health were collected in a consecutive sample of psychotherapy outpatients in routine treatment with at least 1 diagnosed mental health disorder. Results are reported descriptively and supported by exploratory inferential analyses.

**Results:**

Within a total of 812 included outpatients who reported on LLM-CB use for mental health, 245 (30.1%) reported having used LLM-CBs for mental health (once: 69/217, 31.8%; weekly: 118/217, 54.4%; and daily: 30/217, 13.8%). Users were more likely to be younger, female, better educated, students, or unemployed. In total, 61.4% (121/197) have used LLM-CBs in self-reported personal crisis situations, of whom, 67.7% (82/121) found the experience unhelpful. LLM-CB use showed a weak, imprecise association with the number of diagnoses in the included diagnosis groups and was higher for anxiety and eating disorder diagnoses and lower for somatoform disorders. Post hoc multivariable analyses suggest that higher use in eating disorders and lower use in somatoform disorders were attenuated after adjusting for age, while higher use for anxiety disorders persisted. LLM-CBs were generally evaluated positively across various dimensions, especially when self-reported personal crisis situation use was experienced as helpful. Qualitative analysis of an open-ended question on use experience shows LLM-CB use across 4 domains reported in the selected subsample: general psychological assistance, disorder-specific assistance, information assistance, and everyday assistance.

**Conclusions:**

This naturalistic characterization of LLM-CB use in mental health outpatients implies that harms and benefits of LLM-CB use are associated with demographic and clinical profiles. The results suggest that asking about LLM-CB use may be a useful consideration in psychosocial assessment, particularly regarding crisis use, symptom-reinforcing use, and replacement of human support.

## Introduction

### Background

In the 1970s, Weizenbaum [1] developed ELIZA, a rule-based chatbot capable of producing simple conversations with humans in a style that has been compared to a Rogerian psychotherapist. ELIZA identified keywords in a user’s input and responded with questions or queries on the keyword (eg, “I have issues with my *mother*”—“tell me more about your *mother*”). To Weizenbaum’s surprise, users found ELIZA to be comforting, empathetic, and emotionally beneficial [1], despite its mechanical simplicity and inability to understand any of the text inputted or produced.

Sixty years later, chatbots have become a major support tool in everyday human life, including for mental health. Following developments in generative AI and large language models (LLMs) specifically, large language model–based chatbots (LLM-CBs) have been developed, capable of producing natural-sounding human-like text and conversation. A rising number of individuals use LLM-CBs as mental health support tools: available estimates of the proportion of people using LLM-CBs for mental health range from roughly 25% to 50% [2-5], derived from general-population surveys in German and UK samples [2,3] or samples of LLM-CB users in the United States [4,5]. Estimates of LLM-CB use for mental health in clinical populations are, however, largely absent. Furthermore, use data by the LLM company OpenAI show that over a million users of ChatGPT show signs of severe mental distress [6], indicating that LLM-CBs are also used by especially vulnerable individuals.

LLM-CBs may provide several benefits as mental health support tools according to both patients [7-9] and clinical practitioners [7,10,11]. LLM-CBs are often described as nonjudgmental, empathetic, and constantly available [9,12,13]; hence, they may find use as mental health support tools especially when mental health care is limited by resources or logistics [14]. In addition, LLM-CBs are capable of replicating certain psychotherapeutic techniques such as Socratic questioning and cognitive restructuring support [15]. Furthermore, LLM-CBs have shown initial promise as mental health support tools in clinical interventions [16,17], and their role in mental health care is expected to grow [18].

However, perceived benefits of LLM-CBs for mental health have largely been documented through qualitative studies, user reviews, and surveys, with very limited randomized controlled research to validate said benefits. Furthermore, LLM-CBs have also been associated with several types of harms, risks, or limitations that may disturb a safe, ethical implementation as mental health support tools [19]. Those include the generation of false information (ie, hallucinations [20]), sycophantic responses that may validate or reinforce maladaptive patterns [21], inappropriate or harmful content, for example, via jailbreaking [22], data security issues [23], problematic use related to cognitive overreliance or emotional dependence [24], or their limitations regarding high-risk psychiatric cases such as suicidality or AI-associated psychotic episodes [25-27]. In light of such risks and harms, the American Psychological Association [28] and the World Health Organization [29] have released warnings on the use of LLM-CBs as mental health support tools.

Individuals experiencing mental disorders are considered especially vulnerable, and problematic LLM-CB use has been associated with negative mental health in several survey-based studies [30-33]. Especially, unsupervised use of LLM-CBs by individuals with mental health problems has been associated with problematic LLM-CB use later on [34]. Yet so far, systematic, representative data on LLM-CB use by individuals with mental health problems (and patients specifically) remain missing, likely due to recruitment constraints.

### Research Question

This study aims to provide a mixed methods naturalistic characterization of LLM-CB use for mental health among a consecutive sample of mental health patients using descriptive and exploratory inferential analyses. The following research questions were explored:

- Demographic characteristics: What is the prevalence of LLM-CB use for mental health in a clinical outpatient sample, and how is it associated with demographic characteristics (eg, age and sex)?
- Clinical characteristics: How does LLM-CB use for mental health relate to clinical characteristics, specifically for specific International Statistical Classification of Diseases and Related Health Problems, 10th Revision (ICD-10) F-diagnoses and comorbidity?
- Use behavior: What patterns of LLM-CB use are reported by patients, specifically LLM-CB used, use frequency, and use in crisis situations, and how do these vary across demographic and clinical characteristics?
- Use evaluation: How do patients evaluate the experience of LLM-CB use across dimensions of usefulness, understandability, trustworthiness, and emotional understanding, and total evaluations, and how do these vary across demographic and clinical characteristics and use behavior?
- Qualitative: How do patients report their experiences using LLM-CBs for mental health?

## Methods

### Study Design

The data are reported according to the STROBE (Strengthening the Reporting of Observational Studies in Epidemiology) guidelines [35] (see STROBE checklist in Multimedia Appendix 1). Qualitative analysis followed the SRQR (Standards for Reporting Qualitative Research) when applicable [36] (see checklist in Multimedia Appendix 2). A mixed methods cross-sectional analysis in psychotherapy outpatients at a university hospital was conducted.

The study used a convergent mixed methods approach with a predominant quantitative and supportive exploratory qualitative part. Both quantitative and qualitative data were collected within the same routine-care assessment.

### Ethical Considerations

The study was approved by the ethics committee of the Medical Faculty of the University of Duisburg-Essen (26-13042-BO). The study was conducted in accordance with the Declaration of Helsinki. All patients provided electronic informed consent.

### Participants

Participants are routine first-time visitor patients at the psychotherapy outpatient unit at an outpatient clinic for psychosomatic medicine and psychotherapy in a university hospital in the west of Germany. Patients were included if they (1) were of adult age and (2) received an ICD-10 F-diagnosis according to the ICD-10. To reduce bias, recruitment occurred consecutively across 6 months. The recruitment window was October 2025 to March 2026. No formal sample size was calculated, as this analysis is descriptive and exploratory inferential. The sample size reflects available data collected within the recruitment window. Of 1048 patients recorded, 236 patients were excluded due to not responding to the primary question on whether they had used LLM-CBs for mental health before, leading to a total of 812 patients who were included in the analysis. Missingness occurred after providing informed consent.

Standardized mean differences (SMDs) between included and excluded participants are –0.02 (SE 0.07) for age, –0.04 (SE –0.08) for sex, –0.06 (SE 0.07) for anxiety diagnosis, –0.04 (SE 0.07) for depression, –0.21 (SE 0.07) for eating disorder, –0.19 (SE 0.07) for somatoform disorder, +0.11 for trauma or adjustment disorder, and –0.14 (SE 0.07) for comorbidity. To assess whether nonresponse to the primary item was related to LLM-CB use, a logistic model fitted among participants (age, sex, and diagnoses) was used to predict the probability of excluded participants: the model yielded a predicted prevalence of 29.5% of LLM-CB users for mental health in the excluded group, comparable to the 30.1% in the included sample.

Of all included patients, 209 never received psychotherapeutic treatment before, 348 have received it in the past, and 255 currently receive psychotherapeutic treatment.

Following the data collection, all patients proceeded to a clinician-led assessment. The assessment included evaluations of current risk, including suicidal ideation, according to standard clinical practice, and in which, crisis resources and further care were arranged as clinically indicated.

### Measures

Several questions on LLM-CB use experience and behavior were implemented: as a primary question, it was asked whether patients have used LLM-CBs for mental health before (yes/no); this question (“have you used an AI chatbot to consult it about mental health?”) with one example (“ChatGPT”) was used as a filter question for all following questions. The question referred to lifetime use. Participants were not required to identify the system as generative or LLM-based or to provide an example themselves. A broad, unspecified phrasing (“consult about mental health”) was used to capture a wide range of use cases. The generic term “AI chatbot” was used instead of “LLM-based” to prioritize comprehension across patients with varying levels of AI literacy; “LLM” may unlikely be understood by respondents unfamiliar with the technology, which would have introduced differential nonresponse for low-literacy patients. The questions were “how often have you used an AI chatbot to consult it about mental health in the past three months?” (response options: once, once per week, multiple times per week, once daily, and multiple times daily), “which AI chatbot have you used to consult it about mental health?” (response options: open question and multiple answers possible), and “have you ever used an AI chatbot in a personal crisis situation?” (response options: no, yes and helpful, and yes but not helpful) with no examples or definitions of crisis provided in order to reflect subjective self-classification of a subjectively meaningful crisis situation. In addition, 0-100 visual analogue scales (VAS) were used to rate participants’ experiences with LLM-CBs for mental health regarding usefulness (“have you experienced the AI chatbot responses as useful?”), understandability (“have you experienced the AI chatbot responses as understandable?”), feeling emotionally understood (“have you felt emotionally understood by the AI chatbot?”), trustworthiness (“have you trusted the AI chatbot responses?”), and general evaluation (“how would you rate the general experience with the AI chatbot you used for mental health?”). The format choice of VAS with presented values was used based on prior research on patient preference and ease of use [37,38]. Single-item VAS were chosen to minimize response burden for a routine-care baseline questionnaire. Finally, participants were encouraged to share their experience on LLM-CB use for mental health in an open question (“can you tell us more about your (positive/negative) experiences with using AI chatbots for mental health, for example, for what exactly you used them?”).

ICD-10 F-diagnoses were coded according to clinician-assigned ICD-10 diagnoses recorded after specialized clinical interviews. F-diagnoses were grouped according to the following categories: depression (F32.X and F33.X), anxiety (F40.X and F41.X), trauma or adjustment (F43.X), somatoform (F45.X), and eating disorder (F50.X). For reporting, F-diagnoses will be referred to by those terms (eg, depression for F32.X and F33.X diagnoses), as the hospital site clinically focuses on those groups. The group names “anxiety,” “eating disorder,” and “somatoform” were chosen, as those are the respective ICD-10 chapter titles. “Depression” was chosen to specify nonrecurrent (F32) or recurrent (F33) depressive episodes. “Trauma or adjustment” was chosen post hoc as the name, as the 2 predominant diagnoses within this group in the current sample were posttraumatic stress disorder (F43.1) and adjustment disorder (F43.2). Comorbidity count was coded by adding the number of ICD-10 F-diagnoses mentioned earlier.

### Data Analysis and Availability

As a systematic empirical overview on this topic remains sparse, this analysis is conducted as descriptive and exploratory inferential to depict the data (prevalence, demographic and clinical correlates, use patterns, and patient evaluations) on LLM-CB use for mental health among patients with mental health problems. No a priori hypotheses are specified. Data are analyzed using R (version 4.5.0; R Foundation for Statistical Computing). For descriptive data depiction, total numbers and percentages or mean, median, IQR, and SD are used. Age is analyzed continuously and categorized into age bins (10-year steps) for descriptive purposes. Frequency of use is collapsed into 3 tiers (one time, weekly, and daily) for parsimony and to keep relevant cells sufficiently large while keeping relevant use frequency groups (one time, weekly, and high-frequency). Cumulative diagnosis thresholds are computed at each integer level. For exploratory inferential analysis on the magnitude and precision of observed statistical effects, odds ratios (ORs), Cohen *d*, and Spearman ρ are used with *P* values. Wald CIs are reported with 95% range. CIs are reported as precision estimates and null-exclusions (ie, CIs crossing boundary thresholds), and intervals are interpreted in terms of the range of values compatible with the data. Inferential results are not presented as confirmatory and instead as exploratory and hypothesis-generating. Analyses required a valid response of the primary item (LLM-CB use for mental health), and patients not answering it were excluded. All subsequent analyses were conducted on an available-case basis, using all patients with valid data on the variables involved. A number of included cases for each relevant item are named in the Results section whenever the respective outcome is reported. Benjamini-Hochberg (BH) adjustments for controlling the false positive rate at 0.05 false discovery rate (FDR) were selected over family-wise procedures for the 5 diagnosis subgroups. Because patients may carry multiple diagnoses, the 5 tests are positively dependent; as BH retains FDR control under positive regression dependency, it was chosen for the adjustment procedure. Exploratory subgroup analysis is conducted based on relevant groups (ICD-10 F-diagnoses, frequency tier, and self-reported personal crisis situation experience).

For the analysis of the responses to the open-ended question of LLM-CB use experience, a qualitative content analysis including both inductive and structuring elements was conducted on open-ended patient responses on LLM-CB use in order to systematically reduce and categorize qualitative data while preserving meaning, aligning with established standards in qualitative mental health research [39-41]. The qualitative part was situated within a pragmatic, descriptive mixed methods orientation. Qualitative data analysis was conducted by 2 independent coders (TL and NW) with prior experience in qualitative mental health research in a multistep process. Coders were graduate psychologists and psychotherapists in training and were blind to participants’ diagnostic and quantitative data. The open-ended responses were exported from the quantitative or diagnostic dataset, deidentified, and imported into MAXQDA 2026 for qualitative coding. As responses were collected directly as open-ended text, no transcription was required. First, open coding was independently performed by the 2 coders to generate initial codes at the level of meaningful text segments. Then, codes were compared, discussed, and consolidated into a preliminary coding framework collaboratively by both coders. Iterative comparison procedures were used to refine category boundaries and enhance interpretative consistency. To enhance interpretative validity, discrepancies were resolved through discussion until consensus was found. Responses could receive multiple codes. Coding saturation was not assessed, as the analysis was applied to a fixed corpus of preexisting responses; hence, stopping data collection at saturation is not applicable to this design. The finalized coding framework was then applied to the full dataset. Code frequencies are reported descriptively to illustrate relative prominence of themes. Patient quotations were translated from German to English by a native bilingual English or German speaker.

To assess interrater reliability, a post hoc independent coding was conducted based on the developed categories by both raters (NW and TL) 4 months after the development of the codebook. A total of 33% of randomly selected qualitative responses were individually coded by the raters using the developed subcategories. Results show an agreement of 97.4% and a Cohen κ of 0.67, indicating substantial agreement.

Both coders had backgrounds in psychology and prior experience in qualitative research, which may have shaped their interpretation of participants’ responses. To promote reflexivity throughout the analysis, coding decisions, potential assumptions, and differing interpretations were discussed in an iterative process. Discrepancies between the coders were used to critically reflect on and refine the coding framework.

## Results

### LLM-CB Use Characteristics

#### Demographic Characteristics

Demographic characteristics of the final sample are depicted in Table 1 and Table S5 in Multimedia Appendix 3.

**Table 1:** Demographic data of the patients included in the analysis, stratified by whether they disclosed using large language models (LLMs) for mental health before and in total^a^.

Demographic			LLMs used	LLMs not used		Total
Amount, n (%)			245 (30.1)	567 (69.8)		812 (100)
**Sex, n (%)**			182 (29.1)	443 (70.9)		625 (100)
	Male	35 (19.2)		153 (34.5)	188 (30.1)	
	Female	147 (80.8)		288 (65)	435 (69.6)	
	Other	0 (0)		2 (0.5)	2 (0.3)	
	Not reported	63 (25.7)		124 (21.87)	187 (23)	
**Age (years)**						
	Mean (SD)	34.89 (12.07)		43.74 (13.94)	41.07 (14.00)	
	Median (IQR)	33 (18, 29-53.25)		44 (23, 32-55)	39 (24.25, 25-43)	
Number of F-diagnoses, mean (SD)			1.97 (0.93)	1.85 (0.85)		1.89 (0.88)

^a^Percentages are calculated as valid percent, that is, excluding missing values from the denominator.

Patient flow is depicted in Figure 1. Patient demographics across LLM-CB use for mental health are depicted in Table 1. According to descriptive data, LLM-CB users for mental health (vs nonusers) are younger (mean 34.89, SD 12.07 vs mean 43.74, 13.94 years) and more likely to be female (147/245, 80.8% vs 288/443, 65%), to live with parents (27/164, 16.5% vs 30/398, 7.5%), to have a university degree (48/164, 29.3% vs 86/398, 21.6%) or Abitur (58/164, 35.4% vs 106/398, 26.6%), to be in education (33/164, 20.1% vs 23/398, 5.8%), or to be unemployed (34/164, 20.7% vs 57/398, 14.3%). Meanwhile, users (vs nonusers) are less likely to live with a partner (33/164, 20.1% vs 126/398, 31.7%) or to have a secondary school (Hauptschule) diploma (14/164, 8.5% vs 75/398, 18.8%). Sex and age proportions specifically are also depicted in Figure 2.

**Figure 1:**
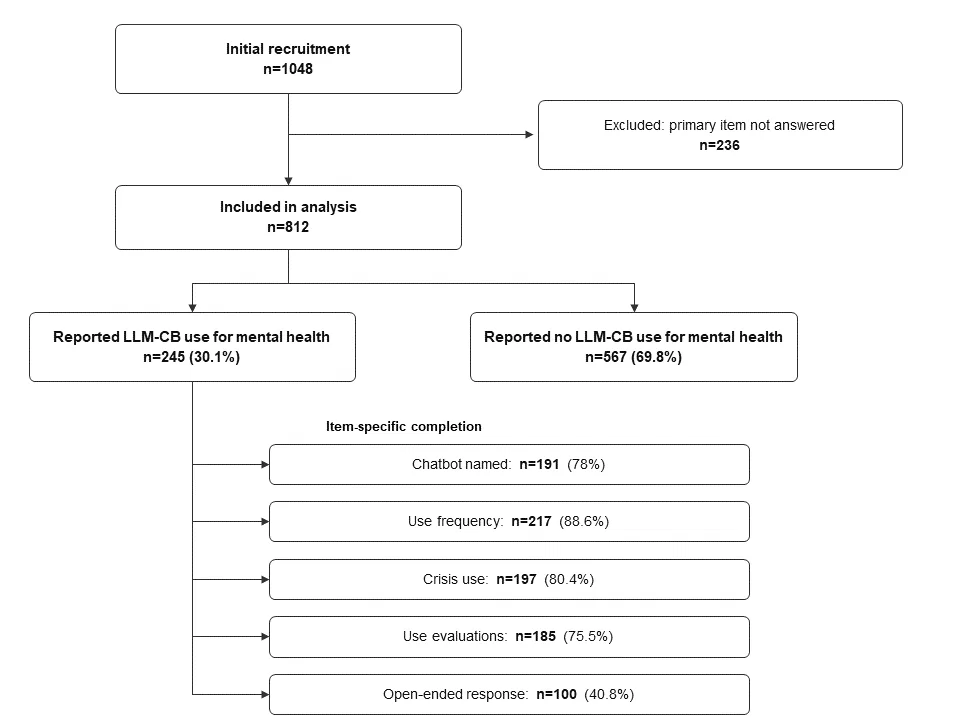
Participant flow and item-level inclusion. LLM-CB: large language model–based chatbot.

**Figure 2:**
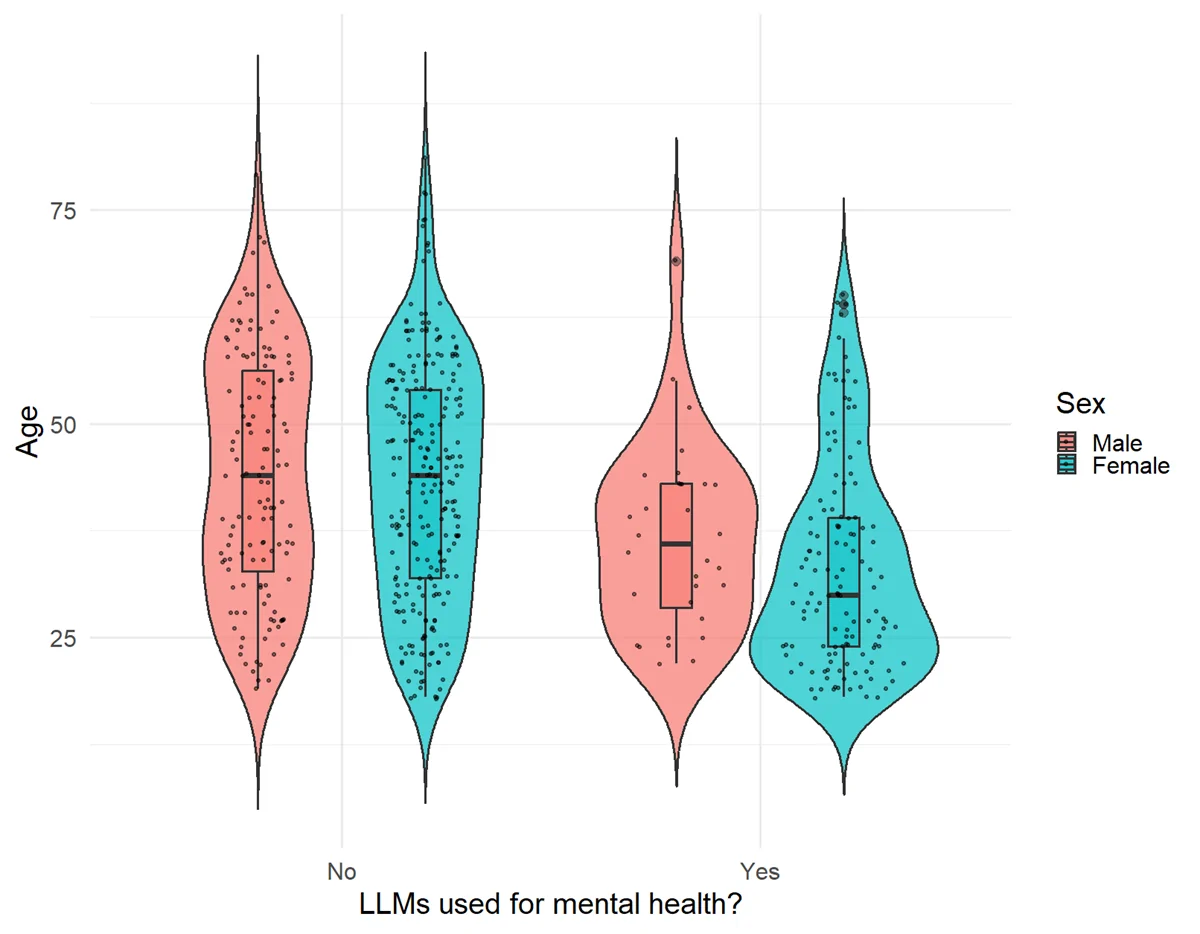
Violin plots and boxplots for age across LLM-CB use for mental health (not used vs used), divided by sex. LLM: large language model; LLM-CB: large language model–based chatbot.

For sex, the odds of using LLM-CB show an OR of 2.18 (95% CI 1.41-3.39; *P*<.001), indicating that female patients have roughly twice the odds to use LLMs for mental health compared to male patients.

For age, violin and boxplots (Figure 2) signal skewness toward younger age in the LLM-CB use group. LLM-CB use across age groups is further depicted in Table 2, showing that 69.8% of LLM-CB users for mental health are younger than 40 years of age (compared to 41.6% for nonusers).

**Table 2:** Total numbers and percentages of large language model–based chatbot (LLM-CB) users and nonusers across age groups.

LLM-CB use	Age group (years)				
	18-29, n (%)	30-39, n (%)	40-49, n (%)	50-59, n (%)	60+, n (%)
					
Yes (n=246)	99 (40.4)	72 (29.4)	37 (15.1)	28 (11.4)	9 (3.7)
No (n=567)	114 (20.1)	122 (21.5)	107 (18.9)	147 (25.9)	77 (13.6)

Finally, patients who have used LLMs for mental health before are younger compared to patients who did not, with an effect size of *d*=0.66 (95% CI 0.51-0.81; t_810_=8.64; *P*<.001), indicating a medium effect. A nonparametric test applied due to the skewedness of the age data shows a rank-biserial correlation of *r*=0.37 (95% CI 0.29-0.44; Wilcox rank sum test *W*=94,932; *P*<.001), corresponding to a probability of superiority of .67 (a random nonuser has a 67% chance to be older compared to a random user).

Of all patients who never received psychotherapy before, 125 (72.7%) have not used LLM-CBs for mental health before, while 47 (27.3%) did. For patients who received psychotherapy in the past, the numbers are 160 (70.5%) for users and 67 (29.5%) for nonusers, and for patients currently in psychotherapy, 106 (68.4%) for users and 49 (31.6%) for nonusers, with no differences between the groups (*χ*^2^_2_=1.0; *P*=.61).

#### Clinical Characteristics

Percentages of ICD-10 F-diagnoses for each condition (LLM-CB use vs nonuse) are depicted in Table 3.

**Table 3:** Percentages of patients who either have or have not used large language model–based chatbots (LLM-CBs) for mental health, stratified by International Statistical Classification of Diseases and Related Health Problems, 10th Revision (ICD-10) F-diagnoses.

LLM-CB use	Anxiety, n (%)	Depression, n (%)	Eating, n (%)	Somatoform, n (%)	Trauma or adjustment, n (%)
Yes	54 (22)	182 (74.3)	75 (30.6)	54 (22	94 (38.4)
No	80 (14.1)	422 (74.4)	118 (20.8)	171 (30.2)	213 (37.6)

^a^Due to multiple diagnoses per patient being possible, percentages add up to values above 100%.

Among the trauma or adjustment group (F43.X), 82 patients were diagnosed with posttraumatic stress disorder (F43.1), 57 with adjustment disorder (F43.2), 23 with other reactions to severe distress (F43.8), and 6 with acute stress reaction (F43.0).

Among the somatoform group (F45.X), 2 patients were diagnosed with somatization disorder (F45.0), 50 patients with undifferentiated somatoform disorder (F45.1), 3 patients with hypochondriacal disorder (F45.2), 4 patients with somatoform autonomic dysfunction (F45.3), 19 patients with persistent pain disorder (F45.4), and 5 patients with other somatoform disorder (F45.8).

For personality disorders, the number of LLM-CB users was 12 compared to 18 nonusers; for psychotic disorders, the number was 2 users versus 3 nonusers; and for substance abuse, the number was 12 users versus 26 nonusers.

ORs of the odds having a specific diagnosis if a patient uses LLMs for mental health compared to nonusers are reported: OR for anxiety disorders is 1.72 (95% CI 1.17-2.53; *P*_adj_=.01), for depression is 0.99 (95% CI 0.70-1.40; *P*_adj_=.97), for eating disorders is 1.68 (95% CI 1.20-2.36; *P*_adj_=.01), for somatoform disorders is 0.65 (95% CI 0.46-0.93; *P*_adj_=.03), and for trauma or adjustment disorders is 1.03 (95% CI 0.76-1.41; *P*_adj_=.97). Hence, 3 diagnosis groups show CIs that do not cross the threshold of 1 after BH correction: anxiety disorders (higher odds for LLM-CB use), eating disorders (higher odds for LLM-CB use), and somatoform disorders (lower odds for LLM-CB use).

LLM-CB use behavior across the number of comorbidities is depicted in Figure 3.

**Figure 3:**
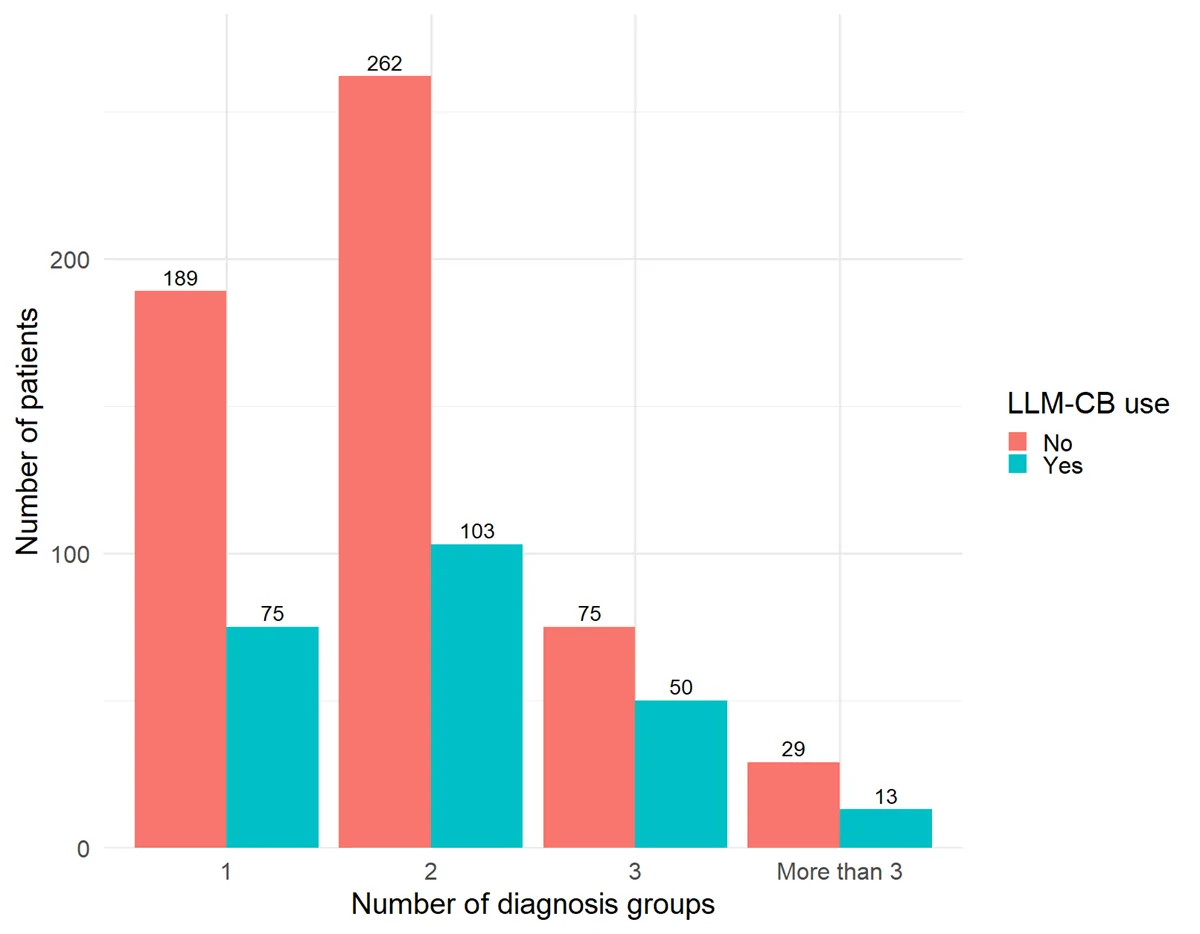
Number of patients across number of ICD-10 F-diagnoses included in the analysis (anxiety, depression, eating disorder, somatoform disorder, trauma or adjustment disorder, personality disorder, substance abuse, and psychotic disorder) stratified by whether they used LLM-CBs for mental health. ICD-10: International Statistical Classification of Diseases and Related Health Problems, 10th Revision; LLM-CB: large language model–based chatbot.

Each additional diagnosis was associated with 1.18 higher odds of ever-use (95% CI 0.99-1.39), a weak positive association whose interval marginally includes null (*P*=.06).

Post hoc multivariable analysis with age, sex, education level, and diagnoses groups as variables shows that sex (OR 1.79, 95% CI 1.15-2.78; *P*=.01) and age (OR 0.62, 95% CI 0.54-0.71; *P*<.001) remained associated with LLM-CB use, as well as anxiety diagnosis (OR 1.75, 95% CI 1.14-2.70; *P*=.001), while eating disorder diagnosis (OR 0.99, 95% CI 0.65-1.50; *P*=.96) and somatoform diagnoses (OR 0.78, 95% CI 0.52-1.19; *P*=.30) were not. Education level was not associated with LLM-CB use for mental health in this model. Sequential adjustments indicate that the crude eating disorder association was primarily attributable to age (adjusted OR 1.31, 95% CI 0.82-2.09), but not to sex (adjusted OR 1.77, 95% CI 1.14-2.76). The same pattern was observed for somatoform disorders (age-adjusted: OR 0.77, 95% CI 0.48-1.23; sex-adjusted: OR 0.62, 95% CI 0.40-0.97). Meanwhile, ORs for anxiety diagnosis increased when adjusting for age and sex (adjusted OR 2.11, 95% CI 1.27-3.50).

### LLM-CB Use Behavior

#### LLM-CB Used

Data show that ChatGPT is used by 62.2% (150/241) of users, distantly followed by 10.7% (26/241) for Gemini. In total, 22.5% (54/241) of patients did not specify the LLM-CB used. DeepSeek and Perplexity were used by 3 of 241 (1.2%) patients each, and Claude, Duck AI, Honestly AI, Meta AI, and Mina AI were used by 1 of 241 (0.4%) patients each. Despite the existence of LLM-CBs specifically designed as mental health tools (eg, Honestly AI), their use in this mental health outpatient sample appears extremely sparse.

Notably, among the 77.5% (187/241) of users who named a system, all named systems were LLM-based, which supports the construct validity of the more generic term “AI chatbot” used for the items.

#### LLM-CB Use Frequency

In total, 217 of 245 (88.6%) patients completed information on use frequency for the last 3 months. Uncollapsed responses are once (69/217, 31.8%), once weekly (41/217, 18.9%), multiple times per week (77/217, 35.5%), once daily (16/217, 7.4%), and multiple times daily (14/217, 6.5%). For collapsed frequency groups, data show that of all patients having used LLM-CBs for mental health in the last 3 months, 31.8% (69/217) used LLM-CBs once, 54.4% (118/217) weekly, and 13.8% (30/217) daily. Use frequency by age is depicted in Figure S1 in Multimedia Appendix 3, and use frequency by sex in Table S1 in Multimedia Appendix 3. Age and sex distributions by use frequency do not show any explicit pattern, other than a slightly higher median age for one-time users compared to more regular users.

Use frequency by diagnosis is depicted in Figure 4.

**Figure 4:**
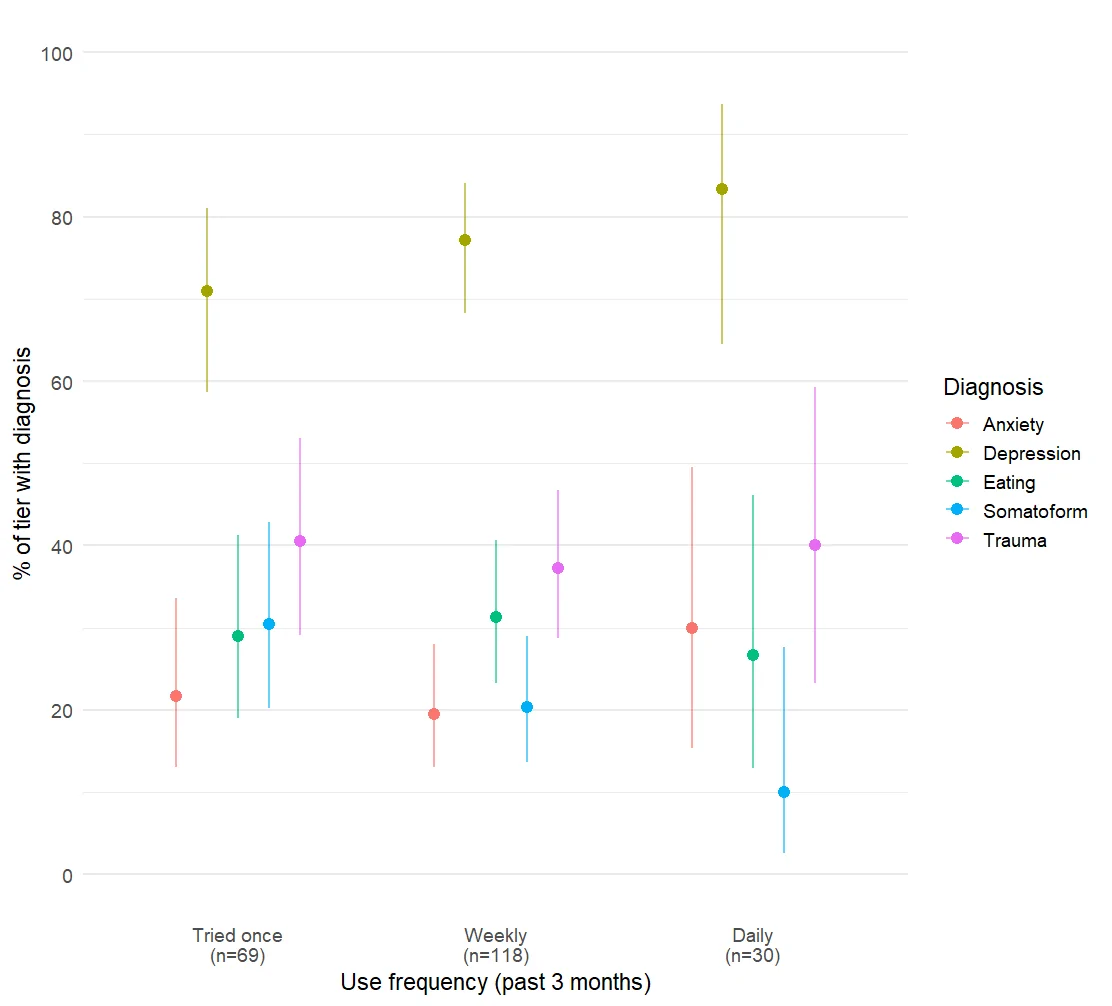
Percentage of patients from recent use frequency tier (once, weekly, and daily), divided by F-diagnosis. Percentages are of patients within each use-frequency tier (once: n=69; weekly: n=118; daily: n=30). Because patients can carry multiple diagnoses, percentages within a tier sum to more than 100%.

Recent use frequency divided by diagnoses shows upward trends of use frequency for depression and anxiety and a downward trend for somatoform disorders.

#### LLM-CB Use in Crisis Situations

In total, 197/245 (80.4%) patients completed information on LLM-CB self-reported personal crisis use. Of those, 61.4% (121/197) reported using LLM-CBs in self-reported personal crisis situations, while 38.6% (76/197) did not. Of those who used LLM-CBs in self-reported personal crisis situations, 67.7% (82/121) found them unhelpful, while 32.2% (39/121) found the use of LLM-CBs in crisis situations helpful. Crisis use experience stratified by age and sex is presented in Figure S2 in Multimedia Appendix 3. Descriptive data suggest that older age and male sex are more associated with no self-reported personal crisis use and experience of crisis use as unhelpful; younger age and female sex are meanwhile associated with crisis use experienced as helpful.

LLM-CB crisis use experience is further shown across categories of use frequency (Table 4) and diagnosis (Table 5).

**Table 4:** Total number and percentages of individuals of different recent frequency large language model–based chatbot (LLM-CB) use groups, divided by self-reported personal LLM-CB crisis situation use.

Use frequency	Not used in crisis, n (%)	Yes, helpful, n (%)	Yes, not helpful, n (%)
Once	40 (62.5)	7 (10.9)	17 (26.6)
Weekly	32 (29.9)	23 (21.5)	52 (48.6)
Daily	4 (15.4)	9 (34.6)	13 (5)

Descriptive data indicate that a higher frequency of using LLMs for mental health is associated with a higher likelihood to use LLMs in self-reported personal crisis situations, with a higher relative increase of helpful compared to unhelpful experience.

**Table 5:** Numbers and percentages of patients with International Statistical Classification of Diseases and Related Health Problems, 10th Revision (ICD-10) F-diagnoses divided by large language model–based chatbot self-reported personal crisis situation use.

Diagnosis	Values, n	Not used, n (%)	Yes, helpful, n (%)	Yes, not helpful, n (%)
Anxiety	46	17 (37)	11 (23.9)	18 (39.1)
Depression	153	60 (39.2)	26 (17)	67 (43.8)
Eating disorder	58	18 (31)	15 (25.9)	25 (43.1)
Somatoform disorder	49	20 (40.8)	6 (12.2)	23 (46.9)
Trauma or adjustment disorder	78	32 (41)	18 (23.1)	28 (35.9)

Descriptive data suggest that some disorder groups (eg, anxiety and eating disorder) are relatively more likely to use LLM-CBs in self-reported personal crisis situations and to experience crisis use as helpful. Meanwhile, other groups (eg, depression and somatoform disorder) may not experience LLM-CB use in self-reported personal crisis situations as sufficiently helpful.

### LLM-CB Use Experience

#### User Evaluations

In total, 185 of 245 (75.5%) patients completed LLM-CB evaluations. Evaluations are shown in Figure 5 for each rating scale asked on how patients perceived or evaluated the LLM-CBs for mental health use (how useful: mean 57.09, SD 22.46; how understandable: mean 71.71, SD 20.79; how trustworthy: mean 49.98, SD 23.74; how emotionally understood: mean 53.57, SD 29.12; and overall rating: mean 58.67, SD 22.03).

**Figure 5:**
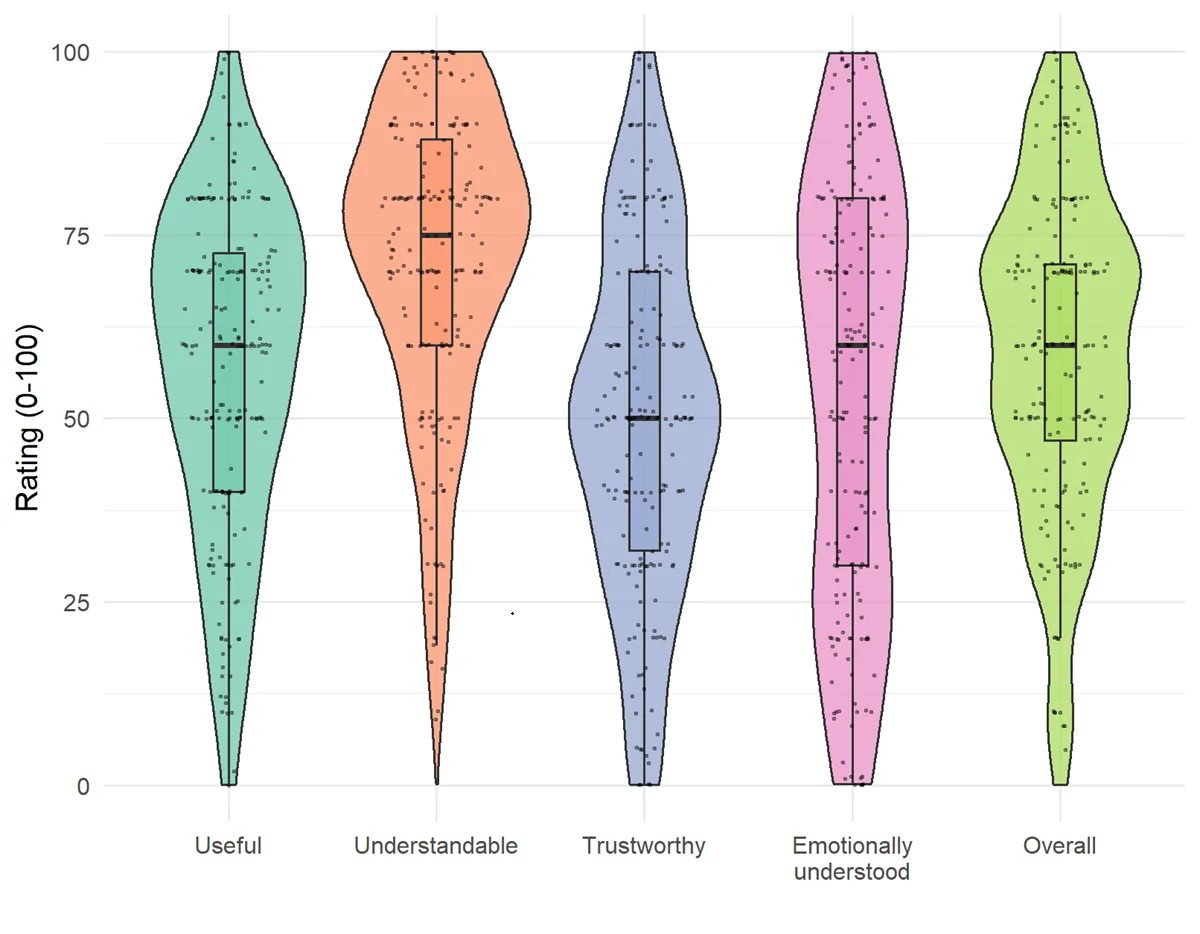
Violin plots and boxplots of patients’ evaluation rating scores on large language model–based chatbot use experience for mental health.

Data suggest that LLM-CB ratings were highest for its understandability and lowest for trustworthiness. While usefulness and overall ratings tended to be positive (above 50), evaluations of feeling emotionally understood suggest a split between high-rating and low-rating individuals.

Spearman ρ correlation coefficients were calculated to investigate intercorrelations between evaluation scales (Table S2 in Multimedia Appendix 3).

Correlations suggest that implemented rating scales generally correlated positively, with coefficients ranging from 0.55 to 0.73, indicating that positive experience of LLM-CBs generalizes across various dimensions of experience.

Furthermore, Spearman ρ correlation coefficients for correlations between recent use frequency and evaluations are 0.21 (95% CI 0.07-0.33; *P*_adj_=.01) for usefulness, –0.03 (95% CI –0.16 to 0.11; *P*_adj_=.70) for understandability, 0.21 (95% CI 0.08-0.34; *P*_adj_=.01) for trustworthiness, 0.15 (95% CI 0.01-0.29; *P*_adj_=.08) for feeling emotionally understood, and 0.22 (95% CI 0.07-0.35; *P*_adj_=.01) for overall rating.

Descriptively, ratings other than understandability and feeling emotionally understood showed a positive association with frequency of LLM-CB use for mental health.

Finally, mean and median evaluations across self-reported personal crisis use experience are depicted in Table S4 in Multimedia Appendix 3.

Data suggest that helpful experience of LLM-CB use in self-reported personal crisis situations was also associated with more positive ratings across scales, while unhelpful use was associated with more negative general ratings. Especially for understandability of LLM-CB outputs, unhelpful experience was associated with lower ratings (54.26) when compared to noncrisis users (71.16), indicating a special association between experience of LLM-CBs as unhelpful in self-reported personal crisis situations and difficulties understanding LLM-CB output. In addition, variation in the group experiencing LLM-CB use in crisis situations as helpful tended to be lower compared to the variations in other groups, indicating that helpful experience of LLM-CBs may be associated with more consistent (positive) experiences.

Finally, evaluations by disorder group are depicted in Table S3 in Multimedia Appendix 3. Values do not indicate different trends in evaluations between disorder groups, except for slightly lower rating tendencies for patients with eating disorder, especially on being emotionally understood.

#### Qualitative Data

##### Overview

In total, 100 of 245 (40.8%) patients completed the open-ended question. Qualitative data analysis on patients’ expressed experience with LLM-CB use for mental health yielded a structured set of categories divided into four domains for the current subsample: (1) general psychological assistance, (2) disorder-specific assistance, (3) information assistance, and (4) everyday assistance. The domains and categories are depicted in Figure 6.

**Figure 6:**
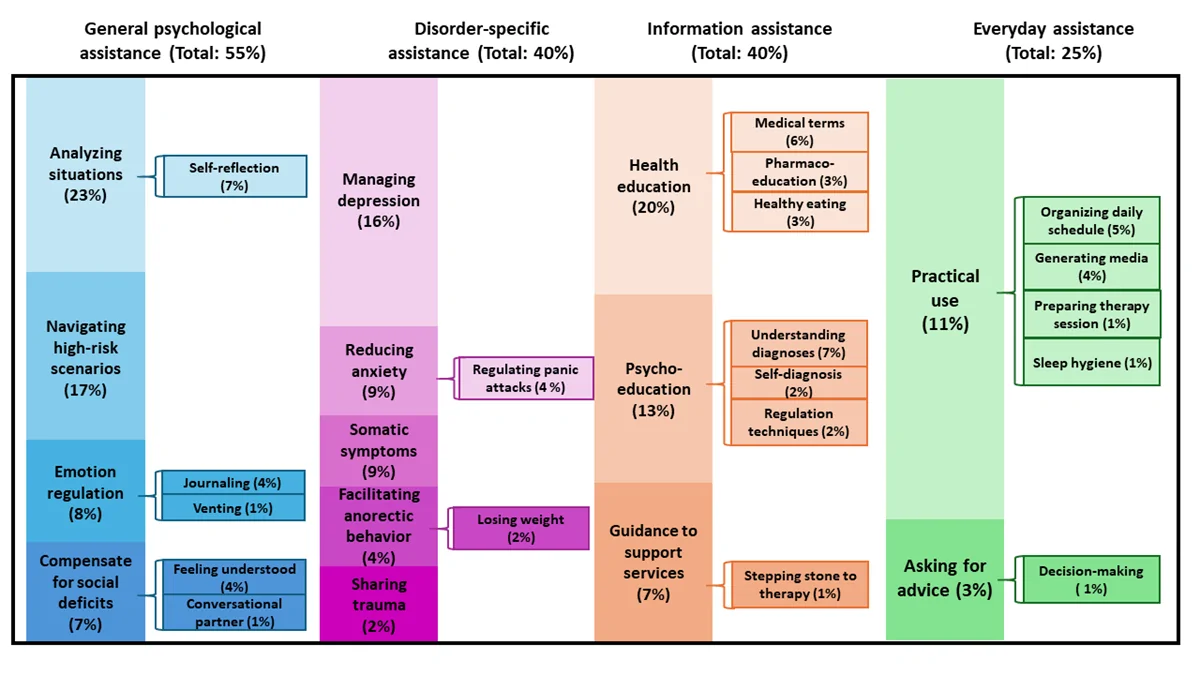
Qualitative responses of patients’ use of large language model–based chatbots for mental health divided into 4 domains and categories. Reported percentages of qualitative data responses are based on participants. Because responses could contribute to multiple categories, percentages may exceed 100%. Category frequencies describe the present subsample and are not prevalence estimates for chatbot users in this clinic or in general.

##### General Psychological Assistance

Patients in this subsample most prominently reported LLM-CB use for general psychological support. Patients reported several uses of LLM-CB for general psychological assistance: to use LLM-CBs to navigate high-risk situations such as acute distress or suicidal ideations (n=17, eg, chatting with a chatbot “when I was planning a suicide attempt, to have someone to talk to”; “it helps in acute situations”), to analyze complex situations such as interpersonal conflicts (n=16, eg, “AI helps to provide an initial overview on one’s situation [...] and to read them structurally”), for emotional regulation such as journaling (n=1, “to write everything down (like a diary)”) or venting (n=1, “to simply vent my spleen”), or to compensate for a lack of social connection (n=7, eg, “helpful when I don’t have anyone to talk to at the moment”). Patients described LLM-CBs as providing immediate mental health support and cognitive structuring. Self-reflection and situational analysis appeared central: patients used LLM-CBs to structure thoughts, analyze interpersonal conflicts, or to obtain alternative perspectives.

##### Disorder-Specific Assistance

The second domain reflects symptom-focused use of LLM-CBs related to specific mental health conditions. Patients frequently reported using LLM-CBs to cope with anxiety (n=9, eg, “to calm myself down during moments of panic and anxiety”), depression (n=12, eg, “to calm down when I ruminate too much”), panic attacks (n=4, eg, “techniques to manage panic attacks”), somatic concerns (n=9, eg, “In a state of anxiety I ask, for example, what increased lymph nodes feel like. Could it be cancer?”), and trauma-related experiences (n=2, eg, “to find closure with my childhood”). For anxiety, patients reported using LLM-CBs for relaxation exercises, grounding techniques, and panic management. Reports of depression-related use included emotional reassurance, rumination management, and sleep-related support. Some patients reported discussing traumatic experiences with LLM-CBs. Patients also reported repeatedly asking LLM-CBs whether certain somatic symptoms could indicate severe illness, reflecting reassurance seeking in anxiety-related and somatoform disorders. For eating disorders, some patients (n=4, eg, “for calorie tracking and calorie burning,” “for plans to lose weight,” and “self-optimization (losing weight), still ongoing, since I have bulimia”) reported using LLM-CBs for calorie calculation or weight loss support, reflecting symptoms of anorexia nervosa.

##### Information Assistance

Patients commonly reported LLM-CB use for psychoeducation (n=13, eg, “I used it once to explain a diagnosis to me and to sort the occurring symptoms”), health education (n=12, eg, about a chronic illness), symptom interpretation for understanding diagnoses (n=7, eg, “I wanted a differentiation between the symptoms” of different diagnoses) or self-diagnosis (n=2, eg, “tool for self-anamnesis for the assessment of my mental distress”), information on medication (n=3, eg, “to estimate my symptoms, effects and side effects of medication”), translation of medical terminology (n=6, eg, “to assess laboratory test results or to explain questions from clinical assessments”), and guidance to support services (n=7, eg, “recommended me to get treated by an experienced physician”). Examples include differentiating symptom profiles, questions on medications and side effects, laboratory values, medication interactions, simplifying medical language, and chronic illness. Patients also reported using LLM-CBs as first-orientation tools toward professional support services. Patients’ behavioral patterns surrounding informational assistance suggest that patients may use LLM-CBs for orientation and explanation on medical topics.

##### Everyday Assistance

The fourth domain involved everyday assistance for practical tasks, such as organizing daily schedules (n=5, eg, “it was also very helpful for structuring my daily routine”), preparing therapy sessions (n=1, “advice for diagnostic clarification when visiting the doctor”), generating media such as texts (n=4, “to generate documents, letters, etc.”), and decision-making support (n=3, eg, asking “for advice very often”). Patients described LLM-CBs as external organizational aids to structure everyday routine and reduce cognitive overload. Patients reported using LLM-CBs for decision support in both interpersonal and everyday situations.

Responses further reflect ambivalent relationships with LLM-CBs: patients simultaneously perceived the systems as positive and supportive (n=6, eg, “I have been able to discuss my problems a lot” and “it’s neutral, no knick-knack, understandable, to me positive”) and LLM-CBs as viable tools for information and knowledge (n=3, eg, “good support and refers to other websites or support, it’s OK to gain an overview”) and reported awareness of shortcomings (n=7, eg, that the LLM-CB “likes to validate whatever I said before”), particularly due to inaccurate outputs and hallucinations (n=6, eg, “I am skeptical because AI has a high hallucination rate” and “the AI is easy to manipulate with targeted questioning”), dependency risks or compulsive use (n=1, using the LLM-CB “really way too much [...] Then I give in more and more and don’t stop at all”), conflicting opinions on anthropomorphism (n=1, “I find these ‘humanlike’ responses helpful but also difficult”), concerns regarding long-term effectiveness (n=1, “I am aware that AI is not a long-term solution”), and the importance of a human-in-the-loop (n=2, eg, “no AI can replace the knowledge and work of a human”).

Qualitative reports on using LLM-CBs for relaxation and panic management converge with quantitative findings of anxiety being associated with LLM-CB use for mental health. The (age-dependent) association between LLM-CB use and eating disorders complements the qualitative reports on using LLM-CBs for calorie tracking and weight loss support, which provide insight into how LLM-CBs are used in that clinical context. The qualitative reports on symptom checking or medical queries may provide an explanatory framework for lower (age-dependent) association between somatoform diagnoses and LLM-CB use for mental health, which may not sufficiently capture somatically framed use. Qualitative reports on LLM-CB use during acute distress and suicidal ideation, mixed with reports on unhelpful responses, converge with the quantitative findings on self-reported personal crisis use and unhelpful experiences. Finally, the qualitative report shows use cases ranging from precare clinical orientation, use between treatment sessions, to LLM-CB use when support is unavailable. These findings complement the quantitative findings that treatment history is unrelated to use, indicating that the specific use case may differ based on treatment history or stage.

SMDs between qualitative question responders and nonresponders are reported for age (SMD=0.43 (SE=0.13); 37.91 vs 32.8 years), sex (SMD=–0.07 (SE=0.16); 21.5% male vs 18.4% male), diagnosis for anxiety (SMD=–0.12 (SE=0.13); 19% vs 24.1%), depression (SMD=0.02 (SE=0.13); 75% vs 73.7%), eating disorders (SMD=–0.17 (SE=0.13); 26% vs 33.8%), somatoform disorders (SMD=0.32 (SE=0.13); 30% vs 16.6%), trauma or adjustment disorders (SMD=0.02 (SE=0.13); 39% vs 37.9%), and general evaluation (SMD=0.15 (SE=0.14); 60.4% vs 57.12%). In addition, responders were less likely to be one-time users (26% vs 36.4%), though daily use was comparable (14.6% vs 13.2%). The qualitative sample thus represents more older patients, more patients with somatoform disorders, and more regular LLM-CB users for mental health. Because one-time users are underrepresented, the identified domains describe use behavior in more recurrent or habitual use. In addition, percentages of categorized self-reported use are not generalizable to other samples.

## Discussion

### Principal Findings

To our knowledge, this work presents one of the first large-scale naturalistic mixed methods clinical characterizations of LLM-CB use for mental health in a psychotherapy outpatient sample. While the topic of LLM-CB use for mental health has previously been dominated by case reports [42,43], vignette studies [44-46], theoretical concerns [21,27], qualitative interview studies [9,12,47], and survey-based studies in nonclinical samples [30-34], the current research extends knowledge by providing a systematic overview on the characterization of a naturalistic cohort.

Among 812 mental health outpatients visiting a mental health outpatient clinic across 6 months, 30.1% (n=245) reported having done so at least once. LLM-CB users for mental health are more likely to be younger, female, be better educated, still in education, be unemployed, and live with parents, compared to nonusers. Age findings parallel population-level data of LLM-CB use, while sex findings contrast them, with more male users using LLM-CBs in general [48,49]. Higher prevalence of female LLM-CB users for mental health may reflect a higher tendency of help-seeking among female individuals in clinical mental health populations. Similarly, higher education has been one of the strongest predictors of LLM-CB use in population surveys and is also associated with LLM-CB use for mental health in the current study [48,49]. Higher unemployment rates in the LLM-CB user group may reflect substitute use for inaccessible health care among economically challenged patients [14] or by severe functional impairment driving both unemployment and additional help-seeking.

Furthermore, LLM-CB use prevalence was comparable across treatment-naïve patients and those already receiving psychotherapy. This indicates that LLM-CBs are used both for substitutive and complementary purposes. Content analysis can be conducted in the future to further investigate differences between substitutive and complementary use.

### Comparison to Prior Work

Anxiety disorders were associated with increased LLM-CB use even when adjusted for age and sex. There are multiple hypothetical mechanisms underlying associations between anxiety and LLM-CB use: anxiety disorders are associated with increased rumination [50,51], reassurance seeking [52-54], and avoidance [55]. It is possible that LLM-CBs may be used as conversation partners for worries or rumination, as providers of reassurance, or as an avoidance strategy, for example, in the context of social anxiety, which has been associated with problematic and more frequent use of LLM-CBs [31,32]. This study also found higher use frequency and (helpful) self-reported personal crisis use for anxiety disorders compared to other groups, indicating that in this group, LLM-CB use is perceived as positive in a way that increases engagement. However, self-reported helpfulness is not a clear indicator of therapeutic value [54], and LLM-CB use for emotion regulation has been associated with problematic use in past research [34,56]. Whether LLM-CB use for anxiety may risk the reinforcement of unsafe or maladaptive patterns (eg, reassurance seeking and avoidance) can be investigated in future research.

Crude associations indicated higher LLM-CB use and a higher proportion of helpful self-reported personal crisis use in the eating disorder group, although that association was attenuated after adjusting for age. Hypothetical mechanisms associating eating disorder symptoms and LLM-CB use are discussed: eating disorder psychopathology, especially for anorexia nervosa and bulimia nervosa, is characterized by ego-syntonic symptoms, with patients actively resisting interventions that challenge eating behaviors and seeking out content that reinforce disordered eating and report such content and environments as helpful and supportive [57,58]. Even clinically used LLM-CBs for eating disorders may generate harmful content [59,60]. Future research can investigate whether LLM-CBs may be used in a symptom-reinforcing manner such as calorie tracking, meal planning, body image queries, and reassurance-seeking.

The crude association between somatoform diagnosis and lower LLM-CB use was likewise attenuated after adjusting for age. One untested possibility is discussed: patients with somatoform disorder typically show resistance to psychologization of somatic symptoms [61-64] and experience psychological framings as dismissive [61,65]. Future research can investigate whether patients with somatoform disorder may be unlikely to use LLM-CBs for mental health and instead focus on health information and physical symptoms (eg, “Cyberchondria” [66-68]), as reflected in the qualitative data.

Depression was descriptively associated with a higher frequency of use (71% once vs 83.3% daily) and a below-baseline helpful rate in crisis situations. Hypothetical mechanisms underlying the association between depression and LLM-CB use behavior are discussed: in past research, depression was positively associated with problematic LLM-CB use [56] and could predict problematic LLM-CB use at a later time point [34]. According to past research, potential mechanisms leading to increased engagement include use for maladaptive coping or emotion regulation [34], rumination and negative perceptions of self and the world potentially leading to corumination [31,69], and increased social withdrawal and interpersonal substitution [70,71]. Loneliness has been associated with problematic LLM-CB use [72,73], especially as a mediator for mental health symptoms [31]. Future research can investigate whether social interactions with LLM-CBs may reduce short-term distress and loneliness while removing the therapeutic experience of actual social interactions, potentially leading to increased social withdrawal.

While evaluations of LLM-CBs for mental health have often focused on validation and sycophancy [21,74], little investigation has been conducted on whether LLM-CBs sufficiently challenge maladaptive cognitive beliefs expressed by individuals in mental distress. In crisis situations such as suicidality, LLM-CBs often provide inadequate responses [45,75], and LLM-CBs are prone to reduce challenging stances and increase sycophancy via user pushback, jailbreaking, or across multiturn conversations [22,72,74,76-79]. Future research may investigate the trajectory of LLM-CB use in depressive individuals, which factors influence upkeep of LLM-CB use, and changes in LLM-CB behavior relative to maladaptive beliefs over time.

For LLM-CB use, ChatGPT dominates with 62% of users, reporting it as the LLM-CB used for mental health, distantly followed by Google Gemini. These data reflect the general prevalence of ChatGPT in LLM-CB use in population surveys [48,49] and have implications for future clinical research in this context: the majority of patients reporting using LLM-CBs for mental health will specifically use ChatGPT, and research on the association between LLM-CB use and mental health may currently be the most representative when focusing on ChatGPT specifically. However, ChatGPT was given as the example item for LLM-CBs in the question, potentially priming participants for responding with ChatGPT.

Furthermore, around 22% of patients did not name the LLM-CB they use, which may indicate low AI literacy, which would imply that around a fourth of patients using LLM-CBs for mental health may not have a clear concept of what they are using. Alternatively, participants may not have been able to recall the LLM-CB name over an unbounded lifetime frame, or a product was used that does not possess a classifiable name. While some LLM-CBs are developed and designed specifically for mental health (eg, Honestly AI, Woebot, Wysa, and Therabot), they are barely used in the current sample. Mental health–specific LLMs generally show better performance for mental health–related tasks compared to general-purpose models, including improved accuracy on mental health classification, more clinically appropriate responding, and better adherence to evidence-based therapeutic principles [79,80]; however, their strong underrepresentation in the current study shows that they are barely reached for by the relevant clinical group, who instead default to general-purpose consumer LLMs not specifically designed or evaluated for mental health support, such as ChatGPT. In addition, many therapy-focused LLM-CBs are not available in Germany or may be more adapted to English-speaking audiences, reducing use in a German sample. For use frequency in the last 3 months, about a third of patients used LLM-CBs for mental health only once, while about half use them weekly. Hence, a third of LLM-CB have not been habitual users in the past 3 months and may show different patterns in demographic and clinical characteristics: for example, LLM-CB one-time users are slightly older than frequent users and show different patterns across diagnoses (eg, anxiety, depression, and somatoform). This urges the need for a proper conceptualization of the “LLM-CB user” in the research field.

Of all LLM-CB users for mental health, 61% used them in acute self-reported personal crisis situations, and 68% of those found them unhelpful, indicating that patients who use LLM-CB in self-classified crisis situations generally do not find them helpful. In addition, qualitative data further show that 17 of 100 responders used LLM-CBs in acute distress or suicidal ideation, as reported by the selected subsample of responders. Vignette study research shows that LLM-CBs tend to underperform in psychiatric crisis situations, for example, by underestimating suicide risks [46,81,82], failing to align with expert consensus [45], and not consistently escalating to emergency resources or human support [44,75]. Furthermore, “helpful” self-reported personal crisis use does not automatically indicate therapeutic value or safety, and instead may merely reflect short-term reduction of acute distress. However, as crisis situations were self-classified in this study, it is unclear to what degree the numbers reflect actual psychiatric crises. Objective markers of crisis use, such as chatbot outputs, escalation behaviors, symptom changes, or crisis outcomes, were not observed or evaluated in this study. Future research may investigate patients’ use of LLM-CBs in psychiatric crisis situations, potential risks for different patient groups, and how perceived helpfulness is associated with actual therapeutic value and safety. However, such research is difficult, as access to patients in crisis situations is often not possible.

LLM-CB use was recorded by brand name only, without version number. The dominant group, “ChatGPT,” spans various model versions that were available at and before the recruitment window, notably ChatGPT 3.5 to ChatGPT 5.0. In addition, model behavior differs further by subscription tier, memory and customization settings, and voice versus text modality. This constrains interpretation particularly for crisis-related and diagnosis-specific findings, and relevant system (especially safety) behaviors have changed across model updates. Future work may record model version, subscription tier, modality (text vs voice), and approximate date of use.

LLM-CB ratings show a generally moderate-to-positive evaluation. LLM-CBs show the highest ratings in understandability and the lowest ratings in trustworthiness. The rating “emotionally understood” signals a more heterogenous distribution. Given that the scale implies LLM-CBs’ ability to emotionally understand, the heterogeneity may be explained by differences in the tendency to anthropomorphize LLM-CBs. Future research may investigate whether anthropomorphizing, especially the attribution of emotional states onto LLM-CBs, explains this heterogeneous distribution.

While frequent use was associated with more positive evaluation, this may reflect positive experiences, leading to repeated use, familiarization effects, discontinuation of dissatisfied users, dependence or overreliance mechanisms, or other unmeasured factors. Future longitudinal research may investigate the mechanisms underlying this association.

Rating scales showed generally moderate to high intercorrelations, with especially high intercorrelation for trustworthiness and usefulness, which may reflect a general evaluative halo effect.

Self-reported personal crisis use experienced as helpful showed more positive ratings across dimensions, while unhelpful crisis experience showed lower ratings, especially for ratings of understandability (average 54.3 ratings for unhelpful self-reported personal crisis use vs 71.2 ratings for no crisis use). Especially, the differences in understandability may have different underlying mechanisms: failed self-reported personal crisis interactions may retroactively degrade the perceived comprehensibility of LLM-CBs. Alternatively, poor comprehension in acute distress situations may drive experiencing the situation as unhelpful. Future research may investigate the exact mechanisms, as it has important implications for application: if LLM-CBs do not provide comprehensive content, especially in self-reported personal crisis situations (eg, due to decreased comprehension ability in acute distress), their use in crisis situations may be especially vulnerable in aspects not previously investigated in vignette crisis situation studies.

Finally, qualitative data analysis indicates that LLM-CB use differs between mental health problems and diagnoses. Despite generally promising and supportive use examples (eg, for emotion regulation and cognitive restructuring), some patient reports indicate potentially problematic patterns of LLM-CB use, such as reassurance seeking in somatic symptom anxiety and calorie tracking in eating disorders. Qualitative analysis furthermore shows that LLM-CBs are perceived as particularly useful when human support is not available or accessible, for psychoeducation, and for understanding medical professional language and records. Patients, however, also described awareness of potential risks (eg, inaccurate information and dependency or compulsive use) and expressed skepticism toward the long-term effectiveness of LLM-CB use or their replacement of health care professionals.

The mixed methods design and integration of quantitative and qualitative results highlight several notions: first, that reports on the use of LLM-CBs for mental health by somatoform disorders are restrained by primarily somatic use; second, that a higher (age-dependent) prevalence of eating disorder use of LLM-CBs for mental health presents itself in using LLM-CBs as a tool for calorie tracking or weight loss strategies; third, that the association between anxiety and LLM-CB use that persisted when controlling for age and sex is reflected in use for relaxation or panic management; and fourth, that LLM-CB use spans across treatment context with varying roles.

### Future Directions

The findings indicate that a substantial proportion of patients arriving at a psychotherapy outpatient clinic have already consulted LLM-CBs for mental health. Clinicians are unlikely to be aware of this unless they ask. Although the clinical value is not yet established, brief enquiry on patients’ use behavior may provide potentially relevant information, especially in the context of LLM-CBs, providing potentially misleading information, symptom-reinforcing use patterns, or the development of problematic use. Enquiry domains of interest are reason of use (eg, information, counseling, conversation, symptom discussion, and symptom-reinforcing use), frequency, crisis use, symptom-reinforcing use (eg, social displacement, reassurance seeking, and dieting), replacement of human care, treatment-supportive use, signs of LLM-CB safety failures (eg, incorrect information), and signs of dependency or problematic use. Whether such enquiry alters clinical decision-making or outcomes would require prospective evaluation that can be investigated in future research.

In summary, the results show that mental health in psychotherapy outpatients may interact with LLM-CB use in several clinically relevant ways. The results suggest the advantage for clinical assessment of LLM-CB use with psychotherapy patients, for example, to recognize potentially maladaptive use patterns like reassurance seeking. Furthermore, this study signals that a differentiating outlook on the interaction between LLM-CB use and mental health is necessary, focusing on differences and unique patterns for individual mental health disorders and clinical profiles.

### Limitations

Several limitations restrain the generalizability of this research. Sampled patients stem from a single site focusing on psychosomatic medicine and psychotherapy, skewing toward a higher representation of certain diagnoses (F3, F4, and F5) and comorbidities. Furthermore, the cross-sectional design does not allow any causal inference. Because illness severity was not assessed, it cannot be determined whether diagnosis-level associations reflect diagnostic category or symptom burden. In addition, missing data led to a notable attrition within the sample, leading to potential issues in sampling selection and generalizability. In addition, evaluation VAS items were self-generated single-scale items with no evidence of reliability or validity and should thus not be treated as established psychometric measures. Correlations between VAS items may reflect a general evaluative halo effect. VAS wordings, such as feeling emotionally understood, may be interpreted differently across respondents, as they may imply humanlike abilities (understanding emotions) and may thus measure anthropomorphization of LLM-CBs. In addition, throughout this work, “LLM-CB use” denoted patient-reported use of a system that the patient themselves identified as an AI chatbot. Although an example was provided (“ChatGPT”) and although 77.5% (187/241) of the patients who reported a specific system reported LLM-CBs, verification of the system used remains patient-reported. Model identity, model version, subscription tier, modality, memory, and customization, among other things, were not verified. The exposure is therefore broad and partly patient-interpreted without objective technical classifications; all results about LLM-CB use should be read with that qualification.

Furthermore, items were designed for a descriptive characterization of data and were selected for providing an overview of the demographic and clinical characteristics rather than theoretical constructs; questionnaires on theoretically relevant constructs, such as problematic use or anthropomorphizing, were not used. Future studies could address this limitation by incorporating validated measures of these constructs to better understand their role in users’ responses. Although qualitative reports provide meaningful insight into subjective experiences of LLM-CB use, only 100 of 245 LLM-CB users responded to those, and the sample of patients who responded to the qualitative item differed in age (older) and diagnoses (more somatoform diagnoses) compared to nonresponders. Hence, the representativeness of the qualitative results is limited by shifted demographic and clinical characteristics. In addition, the use frequency tiers “once,” “weekly,” and “daily” encompass various frequency levels within them (eg, once vs 5 times per week for weekly) and do not sufficiently capture participants with different use behavior prior to 3 months ago. Furthermore, estimates derive from a single-site sample with substantial item nonresponse, and intervals reflect sampling variability; hence, hypothesis-directed replication is required to establish the observed inferential associations. Finally, because this is a single-site cross-sectional observational study with substantial item-level missingness, every diagnosis-level, crisis-use, frequency and evaluation result reported here should be read as exploratory and hypothesis-generating.

### Conclusions

To our knowledge, this study provides one of the first naturalistic observational characterization of LLM-CB use for mental health in a large psychotherapy outpatient sample. Approximately 30.1% (245/812) of included outpatients reported having used LLM-CBs for mental health support. The use of LLM-CBs for mental health is associated with demographics (younger age, female sex, and higher or in education), while frequency and type of use is associated with clinical characteristics. General-purpose models, especially ChatGPT, were predominantly used. Patient evaluations were generally positive and intercorrelated across dimensions; however, the majority of patients using LLM-CBs in self-reported personal crisis situations rated the experience as unhelpful. These findings suggest that asking about LLM-CB use may be a useful clinical consideration during psychosocial assessment, particularly regarding crisis use, symptom-reinforcing use, and replacement of human support. Future research may investigate differences in the quality of LLM-CB use across diagnostic groups. Finally, future evaluations of LLM-CB safety should focus on disorder-specific and multiturn interactions to better approximate real-world patient use and extend research to other groups of mental health disorders.

## Data Availability

The datasets generated or analyzed during this study are available in the OSF repository [83]. Qualitative responses were screened for identifiable information independently by 3 raters. To ensure anonymity and deidentification, qualitative responses were decoupled from the quantitative data and randomized; in addition, of the 100 open-ended question responses collected, 12 were not included in the repository because they contained potentially identifiable information (eg, family constellations, dated life events, and combinations of rare diagnoses).

## Appendix

Multimedia Appendix 1 STROBE checklist.

Multimedia Appendix 2 SRQR checklist.

Multimedia Appendix 3 Supplementary figures and tables. Figure S1 depicts the age of participants in violin plots. Table S1 depicts the percentages of patients divided by gender and use frequency. Figure S2 depicts percentages and violin plots of patients divided by crisis situation use, for gender (A) and age (B). Table S2 depicts Spearman's Rho intercorrelations of patients' LLM-CB evaluation ratings. Table S3 depicts LLM-CB evaluation ratings by disorder group. Tabe S4 depicts median and SQR scores of patient's LLM-CB evlauation ratings divided by crisis use group. Table S5 depicts participants' further demographic characteristics (living situation, education, occupatun, income).

## Abbreviations

BH: Benjamini-Hochberg
FDR: false discovery rate
ICD-10: International Statistical Classification of Diseases and Related Health Problems, 10th Revision
LLM: large language model
LLM-CB: large language model–based chatbot
OR: odds ratio
SMD: standardized mean difference
SRQR: Standards for Reporting Qualitative Research
STROBE: Strengthening the Reporting of Observational Studies in Epidemiology
VAS: visual analogue scale
