# Supplementary material for: Use of Generic Large Language Model Chatbots for Mental Health Issues Among Patients Seeking Psychotherapy: Cross-Sectional Mixed Methods Analysis in a German University Hospital Outpatient Clinic
Source: JMIR AI. 2026 Oct 9;5:e104297. doi: 10.2196/104297 (PMC13695003; doi:10.2196/104297)
Supplement: Supplementary file 3 [file ai_v5i1e104297_app3.docx]

**Figure S1.** Age of participants depicted as violin plots and boxplots, divided by use frequency group.

**Table S1.** Percentages of patients divided by use frequency, per gender.

| LLM-CB use frequency | Female | Male |
| --- | --- | --- |
| Once | 80.49% | 19.51% |
| Weekly | 80.00% | 20.00% |
| Daily | 82.61% | 17.39% |

**Figure S2.** Percentage of male and female patients divided by LLM-CB crisis situation use (A), and violin plots and boxplots of age of patients divided by LLM-CB crisis situation use (B).

**Table S2.** Spearman’s Rho intercorrelations of patients’ evaluation rating scores on LLM-CB use experience for mental health. Values in square brackets represent 95% CIs.

| Scale | Useful | Understandable | Trustworthy | Emotionally understood | Overall |
| --- | --- | --- | --- | --- | --- |
| Useful | 1.00 | / | / | / | / |
| Under-standable | 0.57  [0.47, 0.65] | 1.00 | / | / | / |
| Trustworthy | 0.72  [0.64, 0.78] | 0.59  [0.49, 0.67] | 1.00 | / | / |
| Emotionally Understood | 0.55  [0.45, 0.64] | 0.49  [0.38, 0.59] | 0.55  [0.44, 0.64] | 1.00 | / |
| Overall | 0.73  [0.66, 0.79] | 0.63  [0.53, 0.71] | 0.72  [0.65, 0.79] | 0.69  [0.61, 0.76] | 1.00 |

**Table S3.** Means and standard deviations of LLM-CB evaluations across F-diagnosis groups.

| Group | Useful | Understandable | Trustworthy | Emotionally understood | Overall |
| --- | --- | --- | --- | --- | --- |
| F40.X, F41.X | 55.31 (22.95) | 70.95 (21.08) | 47.04 (26.28) | 57.00 (30.31) | 56.77 (23.65) |
| F32.X, F33.X | 57.12 (22.0) | 72.34 (19.84) | 50.01 (23.27) | 54.90 (28.30) | 59.07 (21.70) |
| F50.X | 54.93 (26.66) | 67.95 (25.59) | 48.16 (26.84) | 47.43 (29.29) | 57.52 (23.96) |
| F45.X | 59.98 (20.07) | 77.42 (16.92) | 49.87 (23.74) | 57.73 (28.63) | 60.39 (21.98) |
| F43.X | 56.64 (22.37) | 71.11 (21.14) | 48.31 (21.55) | 55.04 (29.07) | 60.01 (20.83) |

**Table S4.** Mean (SD) and median (IQR) scores of patients’ evaluation rating scores on LLM-CB use experience for mental health across LLM-CB crisis situation use.

| Crisis use | Helpful | Understandable | Trustworthy | Emotionally  Understood | Overall |
| --- | --- | --- | --- | --- | --- |
| No  Mean (SD):  Median (IQR): | 52.56 (22.5)  52 (40-80) | 71.16 (20.0)  75 (60-86) | 44.38 (25.0)  49 (30-60) | 42.30 (28.9)  44 (20-62) | 50.64 (22.4)  50 (36-70) |
| Helpful  Mean (SD):  Median (IQR): | 69.07 (15.4)  70 (60-80) | 79.57 (14.3)  80 (70-90) | 59.58 (19.2)  60 (50-78) | 70.95 (22.0)  76 (60-89) | 71.64 (15.0)  70 (60-80) |
| Not helpful  Mean (SD):  Median (IQR): | 44.29 (24.1)  50 (30-66) | 54.26 (25.0)  55 (36-73) | 40.21 (23.3)  40 (28-50) | 36.79 (25.5)  30 (18-58) | 44.40 (19.4)  47 (31-53) |

**Table S5.** Home, education, occupation, and income characteristics of the included sample.

| Living situation, n (%)  Alone:  With partner:  Alone with kids:  Partner and kids:  With parents:  Institution:  Other:  Not reported: | 164  53 (32.3%)  33 (20.1%)  11 (6.7%)  28 (17.1%)  27 (16.5%)  1 (0.6%)  11 (6.7%)  81 | 398  136 (34.2%)  126 (31.7%)  24 (6.0%)  69 (17.3%)  30 (7.5%)  2 (0.5%)  11 (2.8%)  169 | 562  189 (33.6%)  159 (28.4%)  35 (6.2%)  97 (17.3%)  57 (10.1%)  3 (0.5%)  22 (3.9%)  250 |
| --- | --- | --- | --- |
| Highest degree, n (%)  University:  Abitur:  High school (Realschule):  Secondary (Hauptschule):  Special needs school:  No degree:  Other:  Not reported: | 164  48 (29.3%)  58 (35.4%)  37 (22.6%)  14 (8.5%)  0 (0.0%)  3 (1.8%)  4 (2.4%)  81 | 398  86 (21.6%)  106 (26.6%)  105 (26.4%)  75 (18.8%)  4 (1.0%)  11 (2.8%)  11 (2.8%)  169 | 562  134 (23.8%)  164 (29.2%)  143 (25.4%)  89 (15.8%)  4 (0.7%)  14 (2.5%)  15 (2.7%)  250 |
| Occupation, n (%)  Self-employed:  Family care:  Civil servant:  White-collar:  Blue-collar:  Other (employed):  Homemaker:  Unemployed:  Retired:  Disability pension:  In education:  Other (unemployed):  Not reported: | 164  5 (3.0%)  0 (0.0%)  2 (1.2%)  69 (42.1%)  2 (1.2%)  1 (0.6%)  6 (3.7%)  34 (20.7%)  2 (1.2%)  7 (4.3%)  33 (20.1%)  3 (1.8%)  81 | 398  14 (3.5%)  3 (0.8%)  13 (3.3%)  199 (50.0%)  15 (3.8%)  8 (2.0%)  9 (2.3%)  57 (14.3%)  19 (4.8%)  13 (3.3%)  23 (5.8%)  25 (6.3%)  169 | 562  19 (3.4%)  3 (0.5%)  15 (2.7%)  268 (47.7%)  17 (3.0%)  9 (1.6%)  15 (2.7%)  91 (16.2%)  21 (3.7%)  20 (3.6%)  56 (10.0%)  28 (5.0%)  250 |
| Monthly net income in €, n (%)  0-500:  501-1000:  1001-1500:  1501-2000:  2001-2500:  2501-3000:  3001-3500:  3501-4000:  4001-4500:  Over 4500:  Prefer not to disclose:  Not reported: | 124  14 (11.3%)  22 (17.7%)  23 (18.5%)  13 (10.5%)  18 (14.5%)  11 (8.9%)  2 (1.6%)  4 (3.2%)  0 (0.0%)  3 (2.4%)  14 (11.3%)  121 | 319  26 (8.2%)  38 (11.9%)  38 (11.9%)  35 (11.0%)  57 (17.9%)  39 (12.2%)  15 (4.7%)  7 (2.2%)  4 (1.3%)  12 (3.8%)  48 (15.0%)  248 | 443  40 (9.0%)  60 (13.5%)  61 (13.8%)  48 (10.8%)  75 (16.9%)  50 (11.3%)  17 (3.8%)  11 (2.5%)  4 (0.9%)  15 (3.4%)  62 (14.0%)  369 |

*Note*. Percentages are calculated as valid percent, i.e., excluding missing values from the denominator.
